# Supplementary figures and images for: The structure of a Type III-A CRISPR-Cas effector complex reveals conserved and idiosyncratic contacts to target RNA and crRNA among Type III-A systems
Source: PLoS One. 2023 Jun 23;18(6):e0287461. doi: 10.1371/journal.pone.0287461 (PMC10289348; doi:10.1371/journal.pone.0287461)

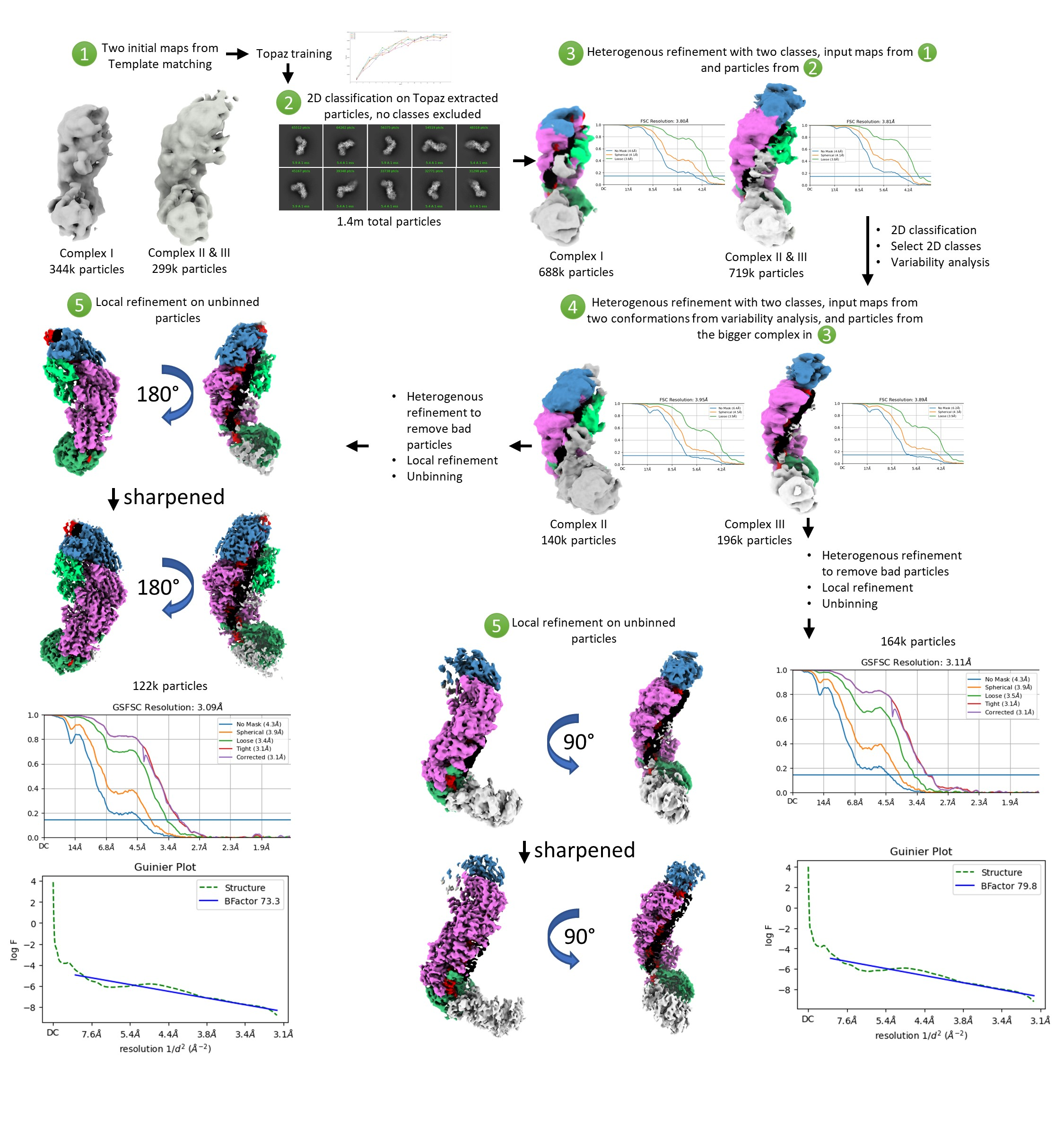

Supplement: S1 Fig — At step 1, two initial maps with different stoichiometries were obtained using template matching. From particles making those maps a set of 5000 particles were used for Topaz training at step 2. Complexes I, II, and III denote the smallest to largest stoichiometries. At step 3, heterogenous refinement was done on the full set of 1.4 million particles. Cas10 is grey, Csm2 is light green, Csm3 is magenta, Csm4 is dark green, Csm5 is blue and target RNA is black. At step 4, the map denoted as complex II and III was further classified with two input maps from variability analysis. Each of the complexes II and III were refined to high resolution with unbinned images at step 5. FSC curves and B-factor plots are included. Due to flexibility of the Cas10, this density is averaged out in the high-resolution refinement of complex II and to a lesser extent in complex III. Csm2 densities are also averaged out at high resolution in complex III because they were under-populated. Complex II was used for the molecular models presented in this manuscript. (TIF) [file pone.0287461.s001.tif]

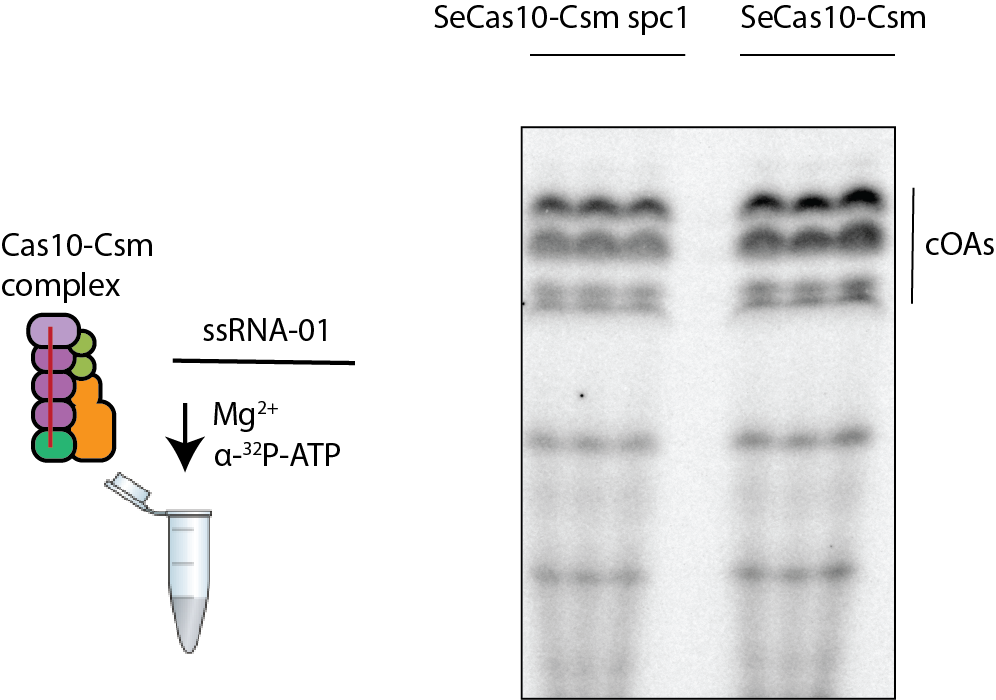

Supplement: S2 Fig — A 24% urea-PAGE gel was used to visualize production of 32P-containing cyclic oligoadenylates produced by the incubation of target RNA (ssRNA-01) and ATP with S. epidermidis Cas10-Csm expressed from the pcrispr spc1 (SeCas10-Csm spc1) plasmid which contains only one spacer gene or SeCas10-Csm expressed from the pcrispr plasmid which contains all three spacer genes found in the S. epidermidis genomic, CRISPR locus. The products from three technical replicates are shown. (TIF) [file pone.0287461.s002.tif]

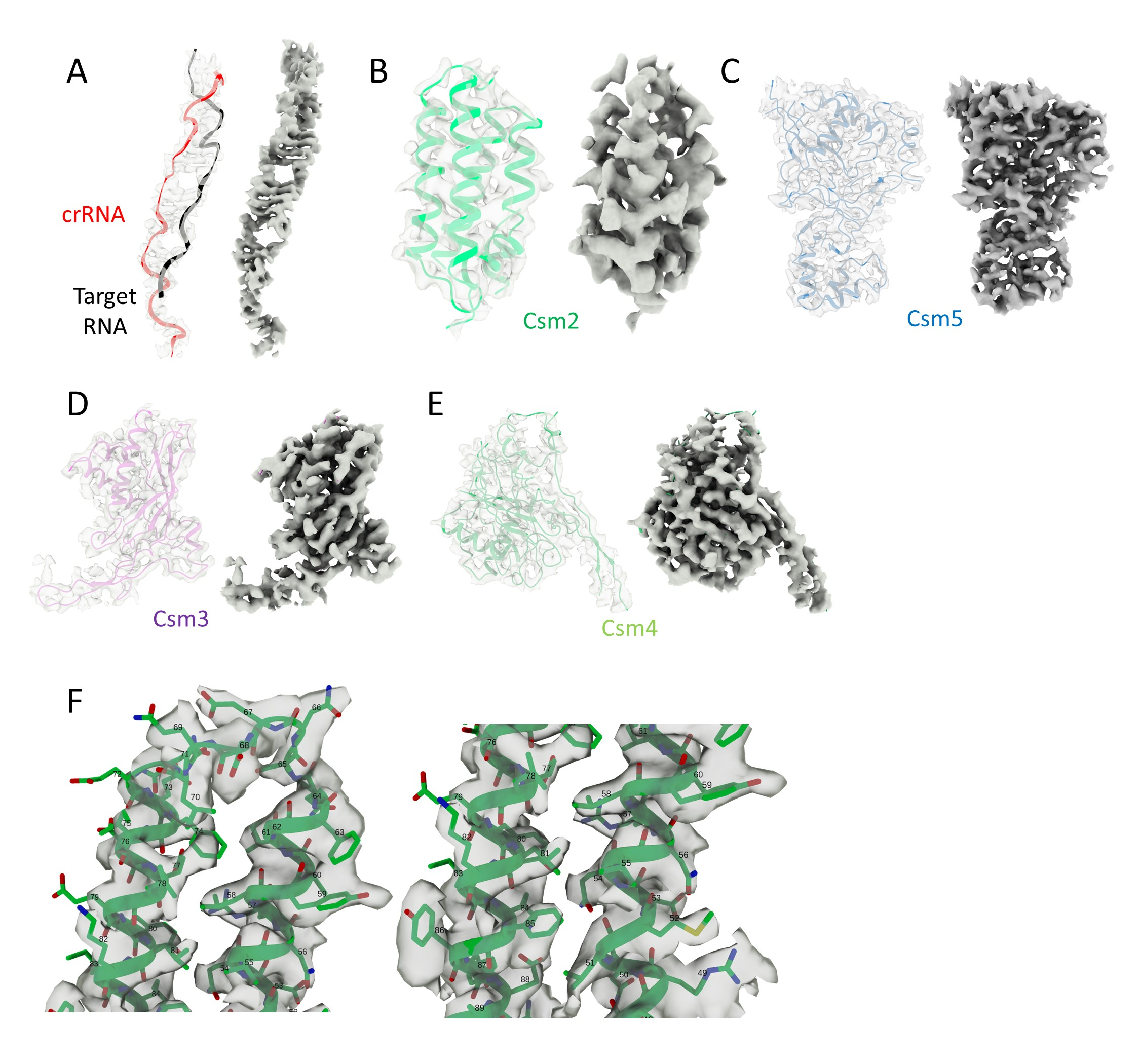

Supplement: S3 Fig — Different subunits of the Cas10-Csm complex are shown in each panel. Each panel contains the density on the right and the model fit in the density on the left. Panel F shows the side chain densities for Csm2 along with the residue numbers. (TIF) [file pone.0287461.s003.tif]

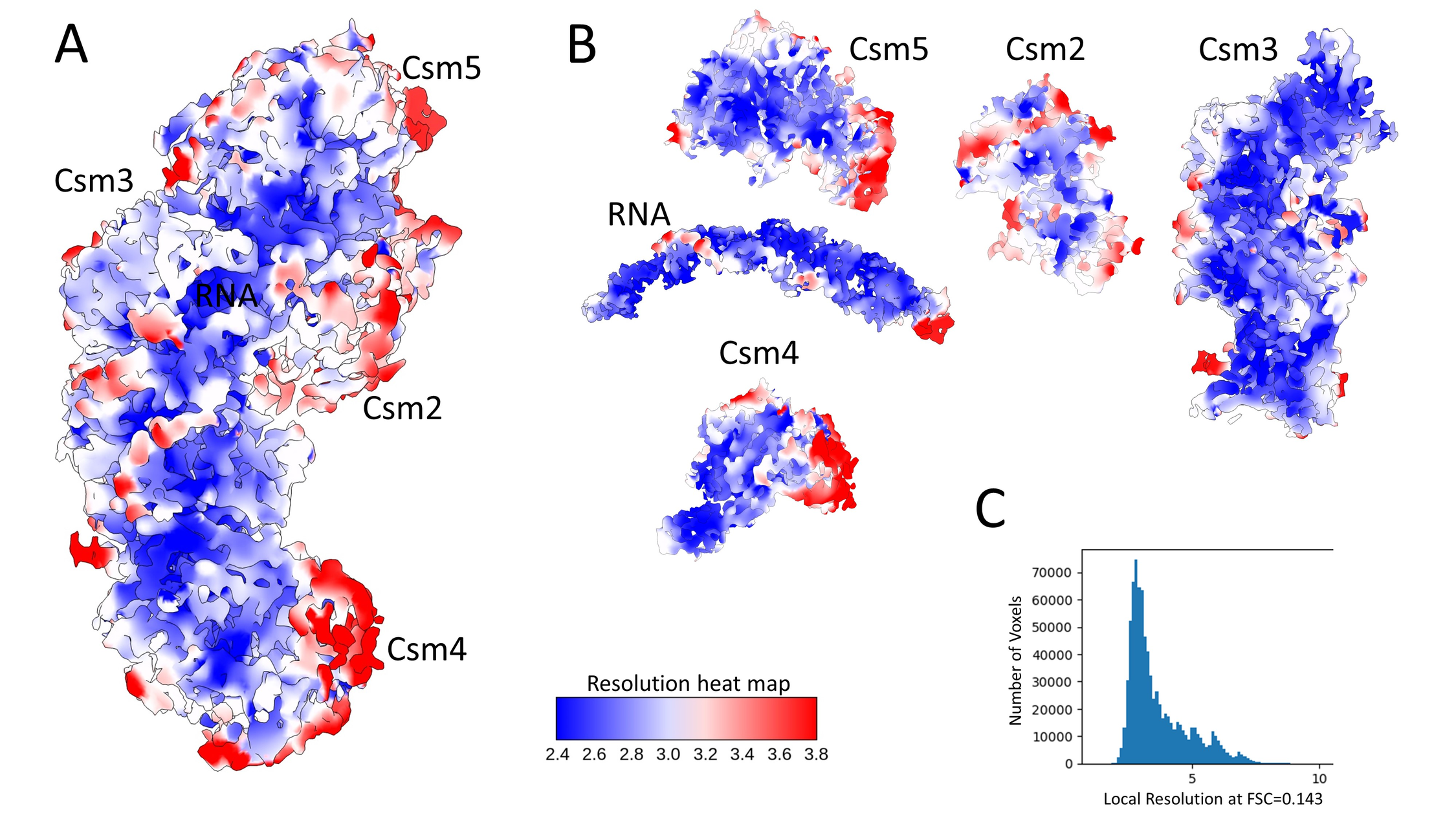

Supplement: S4 Fig — (A) A local resolution plot for the complex of Csm2-5 proteins, crRNA and target RNA. (B) Local resolution plots for the individual proteins and RNA in the complex. The label, RNA, denotes the crRNA-target duplex. (C) A histogram of voxels versus local resolution. (TIF) [file pone.0287461.s004.tif]

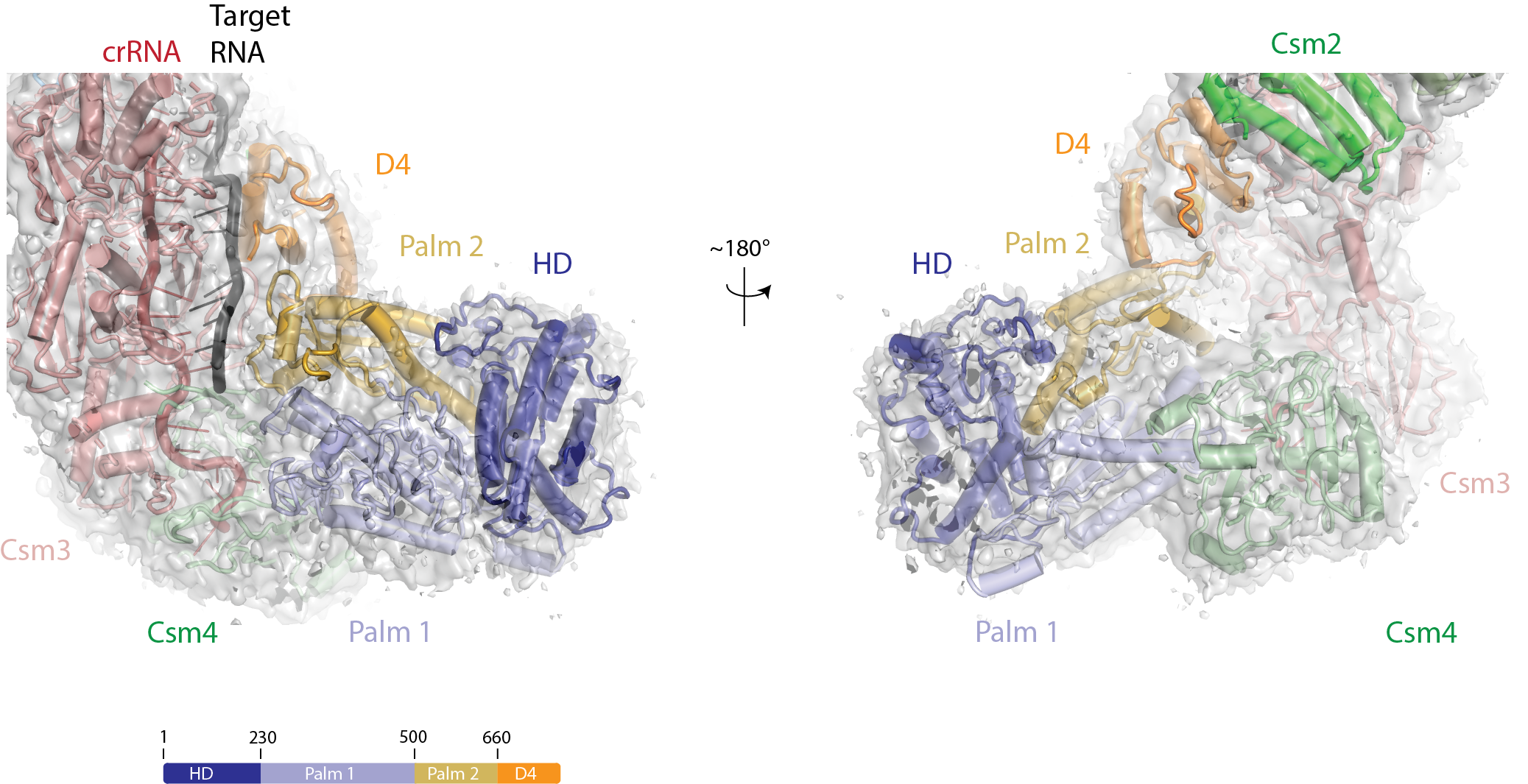

Supplement: S5 Fig — An AlphaFold2 model of S. epidermidis Cas10 was docked into the density given by the map of the 276 kDa Cas10-Csm complex. The map is shown at σ = 2.0. Cas10 is color coded by domain: HD, HD nuclease domain, Palm1, Palm1 polymerase domain 1, Palm 2, Palm polymerase domain 2, D4, domain 4. (TIF) [file pone.0287461.s005.tif]

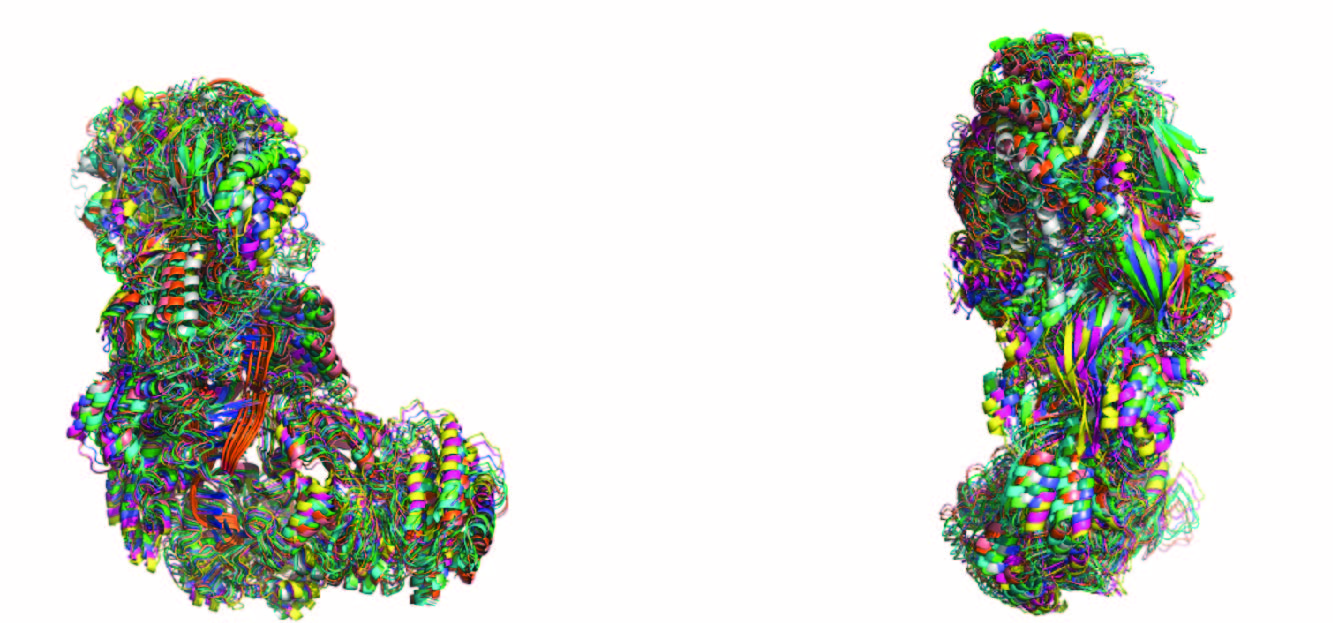

Supplement: S6 Fig — Ten rigid body models calculated in SASREF are shown overlaid with each other; the two views are related by 90 degrees. The overall arrangement and shape of the models are highly consistent. (TIF) [file pone.0287461.s006.tif]

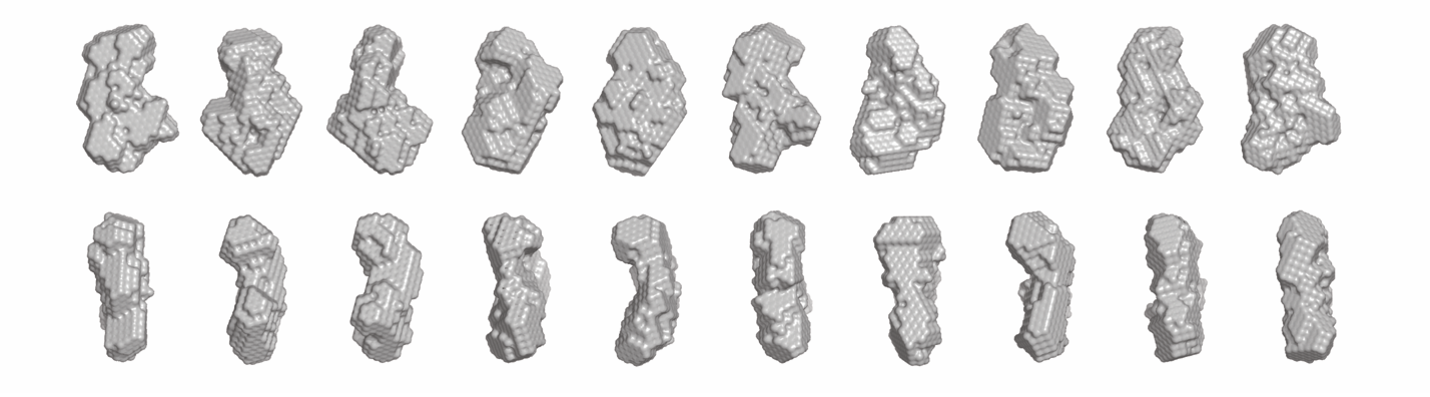

Supplement: S7 Fig — Ab initio models were calculated using DAMMIN, with a Dmax of 160. The upper and lower panels show the same models rotated by 90 degrees. (TIF) [file pone.0287461.s007.tif]

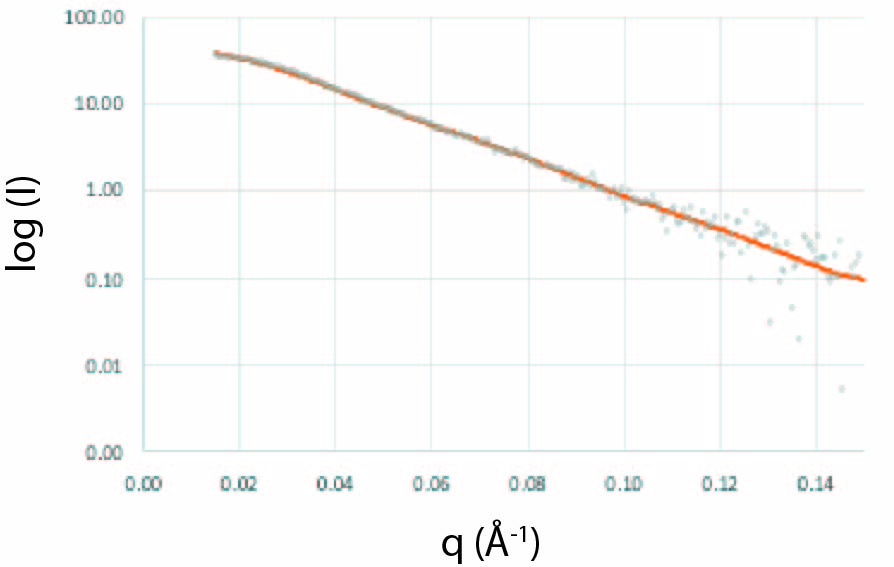

Supplement: S8 Fig — EM models for the 276 kDa complex, the 318 kDa complex, and the 318 kDa complex without Csm2 were used to generate theoretical scattering curves, which were then used in OLIGOMER to deconvolute our experimental SAXS scattering curve. The resulting deconvolution strongly indicated that the 276 kDa complex alone was the best model for the experimental data. The composite fit is shown above as a line with the experimental data as open circles. The Chis for this fit was 1.75. (TIF) [file pone.0287461.s008.tif]

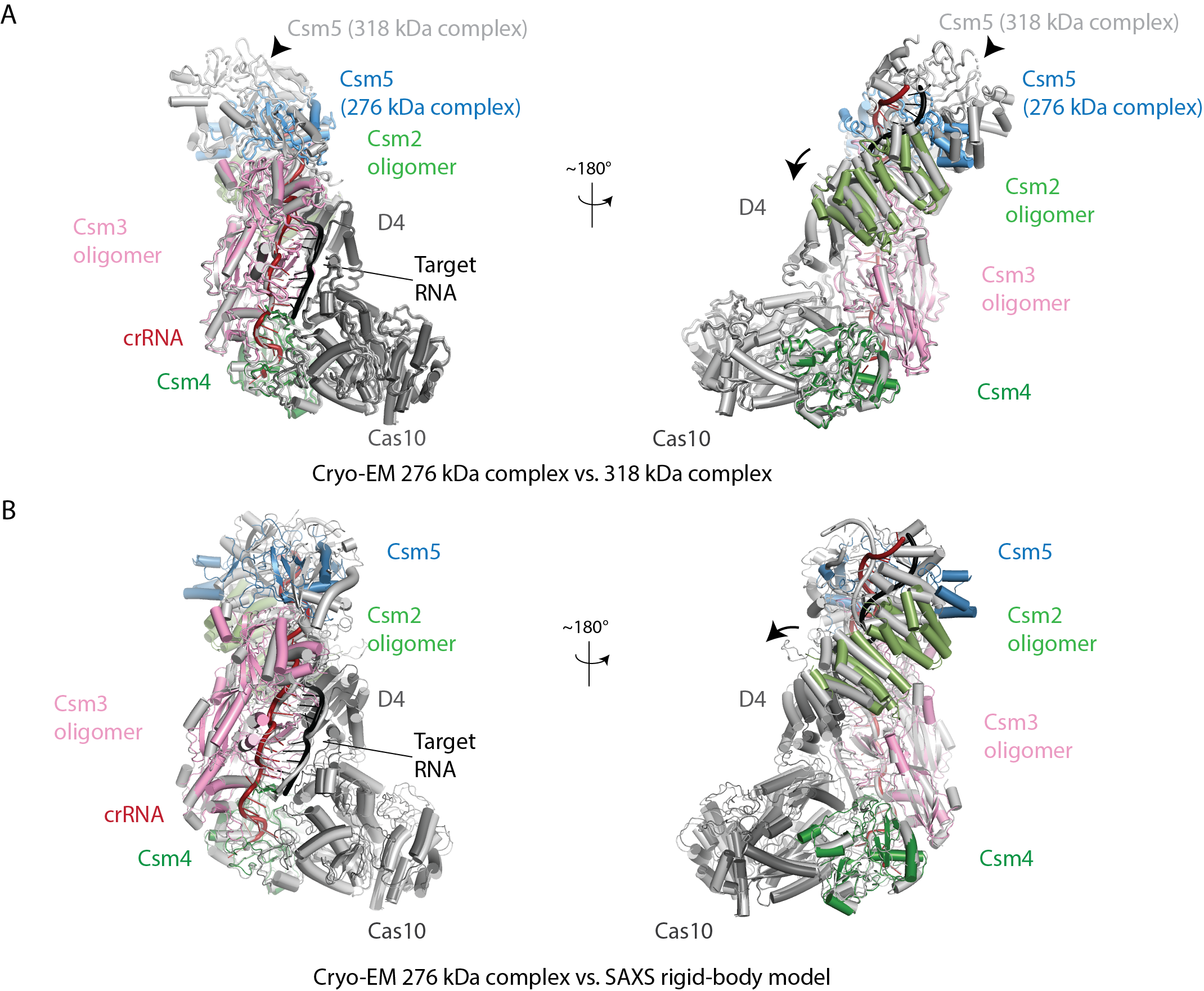

Supplement: S9 Fig — (A) Superposition of the 276 kDa SeCas10-Csm complex (multi-colored, PDB code 8DO6) and the 318 kDa SeCas10-Csm complex (8DO6 chains docked to density, grey) reveals they differ in the number of Csm2 and Csm3 subunits and possess a modest shift of the Csm2 oligomer towards Cas10 (arrow). (B) A superposition of the 276 kDa molecular model (multi-colored, PDB code 8DO6) with a rigid-body model derived by SAXS is shown. Again, a modest shift of the Csm2 oligomer towards Cas10 is observed (arrow). Superpositions were performed using Csm4. D4, refers to Cas10 domain 4, the C-terminal domain of the protein. (TIF) [file pone.0287461.s009.tif]

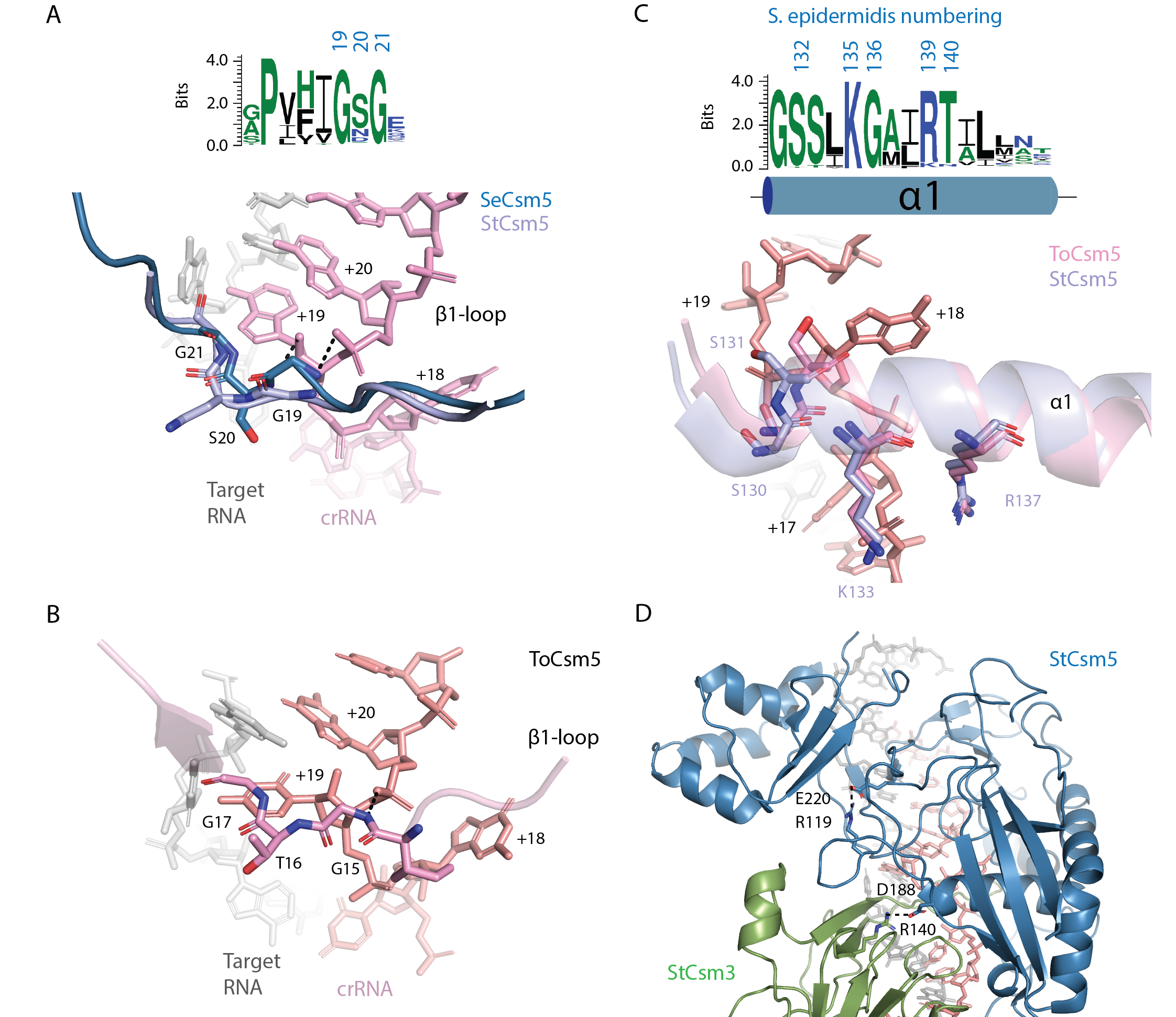

Supplement: S10 Fig — (A) A sharp kink in the peptide backbone of the loop region following β-strand 1 facilitates an interaction with crRNA. A sequence logo depicting the conservation of β1-loop is show above a superposition of S. epidermidis Csm5 (SeCsm5) and S. thermophilus Csm5 (SeCsm5) highlighting similarities in the interactions of the β1-loop with crRNA. (B) The β1-loop of T. onnurineus Csm5 (ToCsm5) interacts with crRNA in a conserved manner. (C) A sequence logo depicting the conservation of helix-α1 which Csm5 interacts with the flipped +18 nucleotide in a similar manner in bacterial and archaeal Csm5 proteins. (D) Csm5 acidic residues required for crRNA maturation in S. epidermidis are conserved in StCsm5 and make similar electrostatic interactions. (TIF) [file pone.0287461.s010.tif]

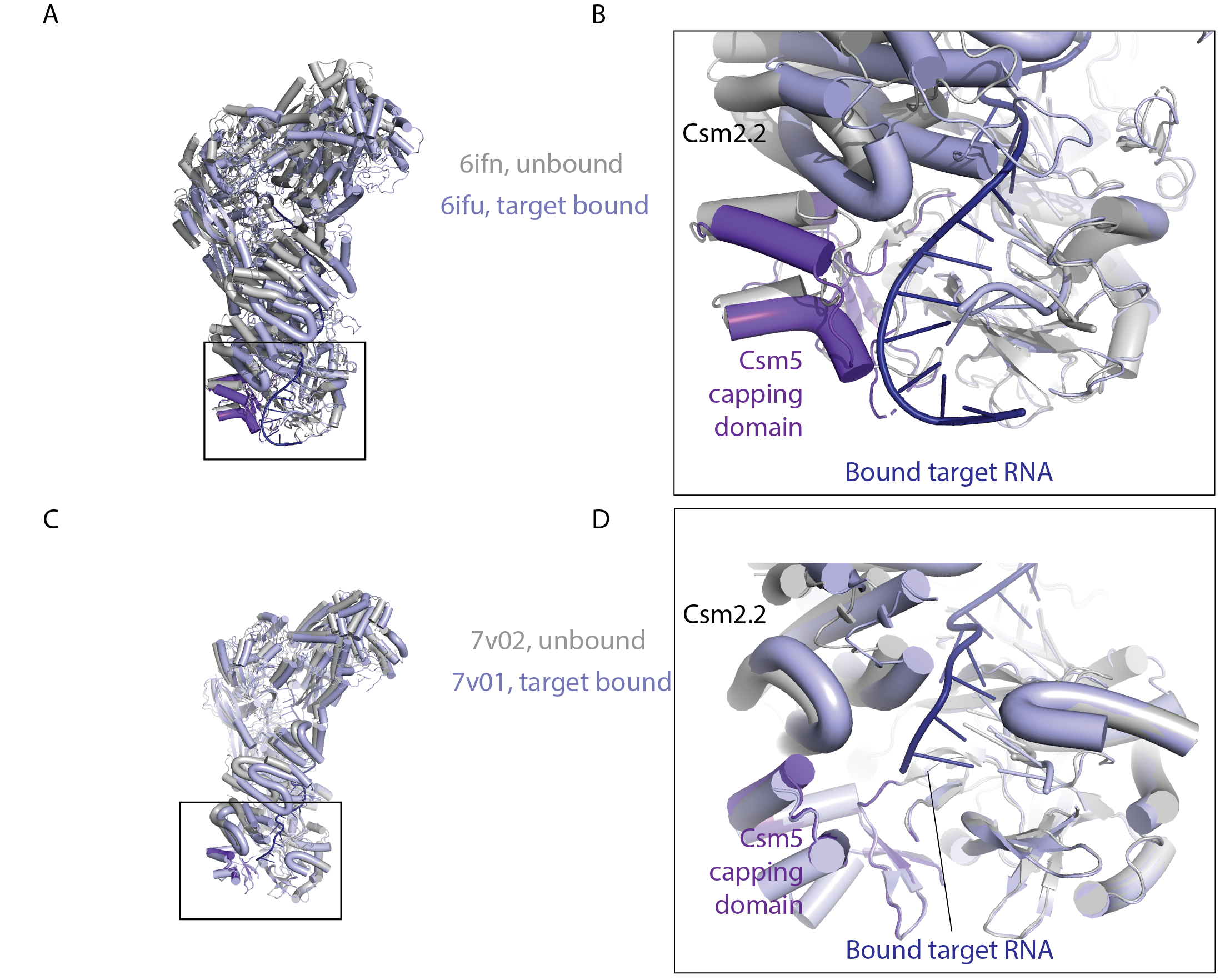

Supplement: S11 Fig — (A) The Cas10-Csm coordinates reported in the target RNA unbound structure, 6ifn, were superpositioned with the target bound structure, 6ifu, using Csm5 residues 111–355 as the basis for the superposition. (B) A detailed view of the boxed area in panel A, shows that the Csm5 capping domain (purple) moves toward the RNA cleft of the complex to interact with bound target RNA influencing the position of neighboring Csm2.2. (C) The Cas10-Csm coordinates reported in 7v02 were superpositioned with 7v01 using Csm5 residues 114–333. The comparison is limited by the fact that 7v01 has a partially cleaved target RNA which is also a non-cognate target, potentially affecting its interaction with the complex. (D) A detailed view of the boxed area in panel C shows a modest movement of the Csm5 capping domain toward bound target RNA. (TIF) [file pone.0287461.s011.tif]
