# Supplementary material for: The structure of a Type III-A CRISPR-Cas effector complex reveals conserved and idiosyncratic contacts to target RNA and crRNA among Type III-A systems
Source: PLoS One. 2023 Jun 23;18(6):e0287461. doi: 10.1371/journal.pone.0287461 (PMC10289348; doi:10.1371/journal.pone.0287461)
Supplement: S1 Table — (PDF) [file pone.0287461.s012.pdf]

Table S1. Oligonucleotides used in the study.

Name	Sequence (5'-3')	Description
F063	TTGCTGCTTAATATATTGCATCATCAAAGATAA ACC	Gibson assembly, p*crispr-spc1*
A010	CTTTGTACTGATGATTTATATACTTCGGCATAC G	Gibson assembly, p*crispr-spc1*
F062	TTATCTTTGATGATGCAATATATTAAGCAGCAA GAG	Gibson assembly, p*crispr-spc1*
L162	CGAAGTATATAAATCATCAGTACAAAGTAAAT CTAACAACACTCTAAAAAATTGTAGATTTTGA ATAAAATACG	Gibson assembly, p*crispr-spc1*
A200	TTGTCAAAAAAAGTGACATATCATATAATCTT GTAC	Sequencing confirmation of p*crispr-spc1*
F111	GCGTCCACGTTTAAATTGTTTGC	Sequencing confirmation of p*crispr-spc1*
ssRNA-01	CUUUGUACUGAUGAUUUAUAUACUUCGGC AUACGUUCUCUAAA	Analog of Nickase mRNA, cognate target for Spc1 crRNA
