# Supplementary material for: The structure of a Type III-A CRISPR-Cas effector complex reveals conserved and idiosyncratic contacts to target RNA and crRNA among Type III-A systems
Source: PLoS One. 2023 Jun 23;18(6):e0287461. doi: 10.1371/journal.pone.0287461 (PMC10289348; doi:10.1371/journal.pone.0287461)
Supplement: S2 Table — (PDF) [file pone.0287461.s013.pdf]

Table S2. Components of the atomic model of SeCas10-Csm (276 kDa complex) bound to target RNA

Name	Uniprot code or sequence (5'-3')	Description
Csm2	Q5HK90	Csm2 protein sequence from *S. epidermidis* RP62A
Csm3	Q5HK91	Csm3 protein sequence from *S. epidermidis* RP62A
Csm4	Q5HK92	Csm2 protein sequence from *S. epidermidis* RP62A
Csm5	Q5HK93	Csm5 protein sequence from *S. epidermidis* RP62A
crRNA	ACGAGAACACGUAUGCCGAAGUAUAUAAAU CAUCAGU	crRNA derived from *spc1* of *S. epidermidis* RP62A
ssRNA-01	CUUUGUACUGAUGAUUUAUAUACUUCGGC AUACGUUCUCUAAA	Analog of *nickase* transcript, synthetic oligonucleotide
