# Supplementary material for: The structure of a Type III-A CRISPR-Cas effector complex reveals conserved and idiosyncratic contacts to target RNA and crRNA among Type III-A systems
Source: PLoS One. 2023 Jun 23;18(6):e0287461. doi: 10.1371/journal.pone.0287461 (PMC10289348; doi:10.1371/journal.pone.0287461)
Supplement: S3 Table — (PDF) [file pone.0287461.s014.pdf]

Table S3. SAXS rigid body components

Rigid body unit	6ifu components (chain ID)	rigid body constraints
1	Csm1 (A) K2 – K654, Csm4 (G), Csm3 (F), crRNA (I) A1 – U13, CTR2 RNA (J) A30 – C34	4.0 A between unit 1 (Csm1 K654) and unit 2 (Csm1 F655), 6.5 A between unit 1 crRNA U13 and unit 2 crRNA C14, 6.5 A between target RNA A30 and unit 2 target RNA G29
2	Csm1 (A) F655 – K757, Csm3 (E), crRNA (I) C14 – U19, CTR2 RNA (J) A24 – G29	6.5 A between unit 2 crRNA U19 and unit 3 crRNA C20, 6.5 A between unit 2 target RNA A24 and unit 3 target RNA G23
3	Csm2 (C), Csm3 (D), crRNA (I) C20 – U25, CTR2 RNA (J) A18 – G23	6.5 A between unit 3 crRNA U25 and unit 4 crRNA C26, 6.5 A between unit 3 target RNA A18 and unit 4 target RNA U17
4	Csm2 (B), Csm5 (H), crRNA (I) C26 – A34, CTR2 RNA (J) A7 – U17	
