# Supplementary material for: The structure of a Type III-A CRISPR-Cas effector complex reveals conserved and idiosyncratic contacts to target RNA and crRNA among Type III-A systems
Source: PLoS One. 2023 Jun 23;18(6):e0287461. doi: 10.1371/journal.pone.0287461 (PMC10289348; doi:10.1371/journal.pone.0287461)
Supplement: S4 Table — (PDF) [file pone.0287461.s015.pdf]

Table S4. Cryo-EM Statistics for Data Collection and Model Quality

Data collection and processing	Cas10-Csm bound to target RNA (8DO6, 276 kDa complex)
Magnification	81,000
Voltage (kV)	300 kV
Electron exposure (e^-/ Å^2)	44.7
Defocus range (µm)	1.0-1.5
Pixel size (Å)	0.846
Initial particles (no.)	1,400,000
Final particles (no.)	122,000
Map resolution (Å)	3.1
FSC threshold	0.143
Map sharpening B factor (Å^2)	73.3
Refinement<br>Csm2-5, target and crRNA	
Model resolution (Å)	3.1
FSC threshold	0.143
Model composition	
Nonhydrogen atoms	12980
Protein residues	1450
RNA residues	61
Bonds (RMSD)	
Bond lengths (Å)	0.005
Bond angles (°)	0.679
Validation	
Molprobity score	2.16
Clashscore	16.50
Ramachandran plot (%)	
Outliers	0.42
Allowed	6.33
Favored	93.25
B factors, mean (Å^2)	
Protein	60.60
RNA	54.33
