# Supplementary material for: The structure of a Type III-A CRISPR-Cas effector complex reveals conserved and idiosyncratic contacts to target RNA and crRNA among Type III-A systems
Source: PLoS One. 2023 Jun 23;18(6):e0287461. doi: 10.1371/journal.pone.0287461 (PMC10289348; doi:10.1371/journal.pone.0287461)
Supplement: S5 Table — (PDF) [file pone.0287461.s016.pdf]

Table S5. Small Angle X-ray Scattering Model Calculation Statistics

**Model type**	**No. of models**	**Average Chi^2**	**Best Chi^2**	**Average NSD or RMSD (in Å)**
rigid body	10	1.724 ± 0.02	1.690	4.6 ± 2.5
ab initio	10	1.479 ± 0.001	1.477	0.603 ± 0.02

Sets of *ab initio* models were calculated from the experimental SAXS scattering profile using DAMMIN. Sets of rigid-body molecular models were calculated from the experimental SAXS scattering profile using SASREF (see Methods for details). Agreement of these models with the SAXS data are indicated by the Chi^2 values (calculated by DAMMIN for *ab initio* models and by SASREF for rigid body models); agreement of these models with each other are indicated with RMSD values (calculated in PyMOL) for molecular models and normalized spatial discrepancy (NSD) values (calculated in SUPCOMB) for *ab initio* models. The average RMSD for molecular models relatively high due to small positional shifts between domains, but the overall shape and architecture remains very similar (see Figure S5).
