# Supplementary material for: The structure of a Type III-A CRISPR-Cas effector complex reveals conserved and idiosyncratic contacts to target RNA and crRNA among Type III-A systems
Source: PLoS One. 2023 Jun 23;18(6):e0287461. doi: 10.1371/journal.pone.0287461 (PMC10289348; doi:10.1371/journal.pone.0287461)
Supplement: S6 Table — (PDF) [file pone.0287461.s017.pdf]

Table S6. Agreement between SAXS *ab initio* models and molecular models

Molecular model type	No. of models	Average NSD	Best NSD
SAXS rigid body models	10	1.8 ± 0.1	1.5909
276 kDa EM	1	1.9 ± 0.1	1.6798
318 kDa EM	1	2.1 ± 0.1	1.9360
318 kDa EM-no Csm2	1	2.5 ± 0.2	2.3224
PDB ID 6IFU	1	2.0 ± 0.1	1.7445

The indicated molecular models were superposed with the set of ten SAXS *ab initio* models using SUPCOMB, and the normalized spatial discrepancies (NSDs) from these superpositions were averaged. For the set of SAXS rigid body models, a comprehensive set of pairwise superpositions was performed.
