# Supplementary material for: The structure of a Type III-A CRISPR-Cas effector complex reveals conserved and idiosyncratic contacts to target RNA and crRNA among Type III-A systems
Source: PLoS One. 2023 Jun 23;18(6):e0287461. doi: 10.1371/journal.pone.0287461 (PMC10289348; doi:10.1371/journal.pone.0287461)
Supplement: S7 Table — (PDF) [file pone.0287461.s018.pdf]

Table S7. CRYSOL fitting statistics

**Models**	**Chi^2**
6ifu	1.568
276 kDa EM	1.591
318 kDa EM	2.657
318 kDa-noCsm2 EM	3.017

The indicated models were used to generate theoretical scattering curves, which were then fit to the experimental SEC-SAXS scattering data using CRYSOL.
