# Supplementary material for: The structure of a Type III-A CRISPR-Cas effector complex reveals conserved and idiosyncratic contacts to target RNA and crRNA among Type III-A systems
Source: PLoS One. 2023 Jun 23;18(6):e0287461. doi: 10.1371/journal.pone.0287461 (PMC10289348; doi:10.1371/journal.pone.0287461)
Supplement: S8 Table — (PDF) [file pone.0287461.s019.pdf]

Table S8. Sequence identities (% identity) among structurally characterized Cas10-Csm complexes.

			Ll	St	To
**Cas10**	Se	Q5HK89	48.1	36.1	23.9
	Ll	L0CEJ3		38.8	23.8
	St	6ig0_A			23.2
	To	B6YWB8			
**Csm2**	Se	Q5HK90	33.3	28.9	14.5
	Ll	L0CFW2		34.6	16.5
	St	6ig0_C			23.3
	To	B6YWB9			
**Csm3**	Se	Q5HK91	53.8	48.1	34.1
	Ll	L0CEA3		53.5	30.9
	St	6ig0_E			32.8
	To	B6YWC0			
**Csm4**	Se	Q5HK92	46.1	31.7	22.4
	Ll	L0CFH1		35.4	21.5
	St	6ig0_G			19.1
	To	B6YWC1			
**Csm5**	Se	Q5HK93	35.2	24.2	12.0
	Ll	L0CG31		25.0	15.5
	St	6ig0_H			9.6
	To	B6YWC5			

Se, *Staphylococcus epidermidis*. Ll, *Lactococcus lactis*. St, *Streptococcus thermophilus.* To, *Thermococcus onnurineus*. Uniprot codes are given for each protein except *S. thermophilus* strain ND03 sequences which are not present in Uniprot. Pairwise sequence alignments were made with Clustal Omega.
